# Sulphate of Iron for Excessive Perspiration

**Published:** 1846-12

**Authors:** 


*Sulphate of Iron for Excessive Perspiration*, prescribed in the following manner, is highly recommended by Professor Lippich:—

℞ Gray cinchona, 30 grammes, or nearly ℥j.

Water 300 " ℥x.

Boil for eight or ten minutes, filter and add
Sulphate of iron, 40 centigrammes, or about gr.vi.
Simple syrup, 30 grammes " ℥j.

Mix, to form a drink, of which a half coffeecupful is to be taken every two hours.—*Jour. de Chimie Médicale.—Ibid.*

